# Electromyographic and Kinematic Comparison of the Leading and Trailing Fore- and Hindlimbs of Horses during Canter

**DOI:** 10.3390/ani13111755

**Published:** 2023-05-25

**Authors:** Lindsay B. St. George, Hilary M. Clayton, Jonathan K. Sinclair, Jim Richards, Serge H. Roy, Sarah Jane Hobbs

**Affiliations:** 1 Research Centre for Applied Sport, Physical Activity and Performance, University of Central Lancashire, Preston PR1 2HE, UKsjhobbs1@uclan.ac.uk (S.J.H.); 2 Department of Large Animal Clinical Sciences, Michigan State University, East Lansing, MI 48824, USA; 3 Allied Health Research Unit, University of Central Lancashire, Preston PR1 2HE, UK; 4 Delsys/Altec Inc., Natick, MA 01760, USA; sroy@delsys.com

**Keywords:** equine, surface electromyography, sEMG, biomechanics, optical motion capture, gait analysis, forelimb, intralimb coordination, lead

## Abstract

**Simple Summary:**

The muscular adaptations that facilitate the differing biomechanical functions of the leading (Ld) and trailing (Tr) limbs during canter in horses remains largely unknown. We conducted the first comparative study of muscle activation and movement within the leading and trailing fore- (F) and hindlimbs (H) during overground canter. Surface electromyography and three-dimensional motion capture data were collected from the right fore- and hindlimbs, as well as the splenius muscle, of ten horses ridden in left- and right-lead canter, when the limbs functioned as TrF/TrH and LdF/LdH, respectively. The TrH is first to make ground contact and exhibited significantly greater gluteal activation than LdH to stabilize the more extended hip joint and to generate greater limb retraction and a strong forward push-off during stance. Then, during TrF and LdH diagonal support, bilateral splenius activation occurred, possibly to counteract downward head and neck movement. The LdF was the last to make contact and was more protracted than the TrF through greater elbow flexion during swing, but triceps activity did not significantly differ between forelimbs. Inter-limb differences in movement and muscle activity provide an objective justification for working the horse equally on both canter leads to promote balanced muscular development.

**Abstract:**

This study compared muscle activity and movement between the leading (Ld) and trailing (Tr) fore- (F) and hindlimbs (H) of horses cantering overground. Three-dimensional kinematic and surface electromyography (sEMG) data were collected from right triceps brachii, biceps femoris, middle gluteal, and splenius from 10 ridden horses during straight left- and right-lead canter. Statistical parametric mapping evaluated between-limb (LdF vs. TrF, LdH vs. TrH) differences in time- and amplitude-normalized sEMG and joint angle–time waveforms over the stride. Linear mixed models evaluated between-limb differences in discrete sEMG activation timings, average rectified values (ARV), and spatio-temporal kinematics. Significantly greater gluteal ARV and activity duration facilitated greater limb retraction, hip extension, and stifle flexion (*p* < 0.05) in the TrH during stance. Earlier splenius activation during the LdF movement cycle (*p* < 0.05), reflected bilateral activation during TrF/LdH diagonal stance, contributing to body pitching mechanisms in canter. Limb muscles were generally quiescent during swing, where significantly greater LdF/H protraction was observed through greater elbow and hip flexion (*p* < 0.05), respectively. Alterations in muscle activation facilitate different timing and movement cycles of the leading and trailing limbs, which justifies equal training on both canter leads to develop symmetry in muscular strength, enhance athletic performance, and mitigate overuse injury risks.

## 1. Introduction

In quadrupeds, an asymmetrical gait is one in which the footfalls of one or both contralateral limb pairs occur as couplets, which implies an unequal time interval between left and right footfalls [1]. The limbs contacting the ground before and after the shorter time interval are called the trailing (Tr) and leading (Ld) limbs, respectively [1,2,3]. Canter and gallop are common asymmetrical gaits in horses. When the leading limbs are on the same side of the body in the fore- and hindlimb pairs, the gait is said to have a transverse sequence, whereas a rotary canter/gallop has the leading limbs on opposite side for the two limb girdles [1]. In cantering horses, both the fore (F) and hind (H) limb footfalls occur as couplets, and the order of limb contacts typically follows a transverse sequence [1]. Additionally, movements of the LdH and TrF are synchronized [1,4,5,6,7,8,9]. The footfall pattern is: TrH, LdH-TrF, then LdF [4,5,6,7,8,9]. Based on the leading forelimb, the horse is said to be cantering on the left lead or the right lead [4,5,7,8].

The canter represents an important gait due to its frequent and essential use in equestrian competition and training [4,10]. It is the gait employed during show jumping competition [11], and the development of a quality canter has been described as one of the most important aspects of jump training programmes [12]. Dressage horses perform four variations of the canter (collected, working, medium, extended) as well as the execution of half pass, canter pirouettes, and flying lead changes [8,9]. Furthermore, research has determined that canter is the only trait evaluated during performance testing that is highly correlated with future performance in both dressage and show jumping [13,14]. Despite the importance of this gait for sport horse training and competition, relatively few studies have focussed on canter within the equine biomechanics literature [10]. Of these studies, the differing functional demands of the leading and trailing fore- and hindlimbs during canter have been quantified [4,5,15,16], but the underlying differences in muscle function that facilitate these differences remain largely unknown.

During canter, the leading limb is often referred to as the “swinging” limb and is brought more forward during swing phase, while the trailing limb is generally referred to as the “supporting” or “propelling” limb [4,15]. These definitions have been supported by kinetic [5,15,17] and kinematic [4,16] studies. The “supporting” trailing limbs exhibit the greatest propulsive forces [5,15], with the TrF also experiencing the greatest vertical impulse and vertical loading of approximately 1.5 times the horse’s body weight [5]. The trailing limbs have been reported to show significantly more retraction [4,16], which Back et al. [4] link to their closer orientation to the centre of mass (COM). The trailing limbs also show greater metacarpophalangeal (MCPJ) [4,16] and metatarsophalangeal joint (MTPJ) extension during stance [4] that is associated with greater vertical limb loading [18]. In contrast, the “swinging” LdF and LdH exhibit the greatest braking forces combined with minimal propulsive forces, which is related to their functions of controlling the lowering of the COM during the stance phase and subsequently raising of the COM prior to the suspension phase [5,15]. Significantly greater protraction has been observed in the leading limbs [4,16], which is driven by significantly greater hip and elbow flexion throughout the stride cycle [4,16]. The differences in movement, intralimb timing and coordination, and loading between leading and trailing limbs during canter [4,5,16] indicate that horses should be worked regularly on the left and right canter leads to strengthen the muscles symmetrically and thus reduce the risk of fatigue and injury. However, further research is required to quantify the actions of muscles that facilitate these kinetic and kinematic differences between leading and trailing limbs during canter.

Surface electromyography (sEMG) has been used in several studies to quantify muscle function during walk and trot [19,20,21,22,23,24,25]. However, relatively few studies have used sEMG to study muscle function during treadmill [19,26] or overground [27,28] canter. One study used sEMG to study between-limb differences in muscular function during canter [28]. In this study St. George et al. [28] provided a proof of principle for appropriate sEMG signal processing, developed using the known differences between LdH and TrH loading and movement during canter [4,5,15,16]. Biceps femoris muscle activity, measured using average rectified values (ARV) and integrated EMG (iEMG), was significantly greater in the LdH than TrH. This finding led the authors to suggest that biceps femoris must work eccentrically with greater force to stabilise the hip and stifle joints of the LdH [28], which experience greater limb loading [5]. This study provided preliminary insight into differences in muscle function related to biomechanical changes during canter. However, to our knowledge, no studies have compared both the movement and underlying muscle activation of leading and trailing fore- and hindlimbs within the same horse during overground canter. This information would have real-world applications for equestrians, as understanding the differing neuromuscular demands between leading and trailing limbs during canter could aid in the identification of muscular imbalances and the design of training programs to improve muscular symmetry and athletic performance.

Thus, the aim of this study was to measure and compare muscle function and movement between the leading and trailing fore- and hindlimbs during overground canter using sEMG and three-dimensional (3D) motion capture. In addition, head and neck movement represents an active element of the fundamental gait mechanism during canter [29,30], so we evaluated splenius activation in the context of LdF and TrF movement cycles. Within the forelimbs, it was hypothesized that the TrF, which experiences the greatest vertical limb loading across all limbs [5], would exhibit greater joint flexion/extension during stance and greater muscular activity across the stride cycle than the LdF. Within the hindlimbs, it was hypothesized that greater protraction [4] and vertical limb loading [5], experienced by the LdH, would be associated with greater joint flexion/extension and greater muscular activity across the stride when compared to the TrH.

## 2. Materials and Methods

Ethical approval for this study was obtained from the University of Central Lancashire’s Animal Projects Committee (RE/13/04/SH). Written informed consent was obtained from all horse owners and riders prior to data collection.

### 2.1. Horses and Horse Preparation

Data were collected from 10 riding horses (mean ± SD age: 10.6 ± 2.4 years, height: 160.9 ± 8.0 cm, sex: 5 mares, 5 geldings, breed: 7 Warmblood, 1 Thoroughbred, 1 Irish Sports Horse, 1 Arabian × Welsh Cob). All horses were in work/training at the time of the study and were physically fit. Six (*n* = 6) horses had competed at a minimum level of British Showjumping Foxhunter up to 1.60 m international show jumping classes. Four (*n* = 4) horses had lower-level eventing and show jumping competition experience at jump heights ranging from 0.8 to 1.0 m. Our study focusses on movement and muscle activity within the general riding horse population, so lameness evaluations were not conducted by a veterinarian, and all horses were believed to be sound by their owner/rider. Horses were ridden by their normal rider, each with 14–20 years riding experience and having competed at a minimum level of 1.0 m unaffiliated show jumping. Prior to data collection, horses completed a short warm up of approximately 15 min in walk, trot, and canter at the rider’s discretion. Following warm up, kinematic markers and sEMG sensors were attached over pre-determined anatomical locations and to pre-prepared skin over superficial muscles, respectively. Spherical retro-reflective markers (25 mm diameter) were attached over anatomical landmarks on each horse’s right fore- and hindlimb, as described by St. George et al. [31].

Surface EMG sensors (Trigno, Delsys Inc. Natick, MA, USA) were unilaterally positioned over prepared skin on the right side of each horse to record from the long head of triceps brachii (triceps), middle gluteal (gluteal), vertebral head of biceps femoris (biceps), and splenius muscles. The reader is referred to St. George et al. [31] for detailed descriptions of sensor site locations for each muscle. Prior to data collection, hair was removed from sensor sites and thoroughly cleaned with isopropyl alcohol. A small amount of saline solution was applied to the electrode bars to act as an electrolytic solution before sensors were adhered to prepared skin using a combination of Delsys Adhesive Surface Interface Strips (Delsys Inc., Natick, MA, USA) and strips of double-sided tape [31]. Sensors were positioned on the muscle belly, with the electrodes oriented perpendicular to the underlying muscle fibre direction [32,33].

### 2.2. Equipment Set Up

Eight infrared Qualisys Oqus cameras (Qualisys AB, Goteborg, Sweden) were positioned side-by-side in a linear configuration and an extended calibration was conducted to enable the collection of data from multiple strides. The calibration volume was approximately 8 m in length, and ground poles were placed parallel to and approximately 4.5 m from the cameras to define the optimal capture volume for horse/rider combinations.

### 2.3. Data Collection Protocol

The sEMG and 3D kinematic data were collected from the right side of the horse at 2088 Hz and 232 Hz, respectively, during ridden canter trials. Unilateral sEMG and kinematic data were collected during right- and left-lead canter in a random order. The right forelimb and hindlimb functioned as LdF and LdH during right lead canter and as TrF and TrH during left lead canter. Kinematic and sEMG data were collected synchronously using an external trigger system (Delsys Trigger Module, Delsys Inc. Natick, MA, USA) and Qualisys Track Manager software (version 2018.1, Qualisys AB, Goteborg, Sweden).

A ridden static trial was initially recorded with each horse standing in the centre of the optimal capture volume. Canter trials were then collected, with each horse being ridden through the optimal capture volume (adjacent to the placing poles) at their preferred canter velocity. A minimum of six canter trials were collected for each horse, with three trials collected from randomised left- and right-lead canter. A trial was deemed successful when the horse maintained the canter and correct canter lead and remained within the optimal capture volume.

### 2.4. Data Processing and Analysis

#### 2.4.1. Kinematic Data Processing and Analysis

Kinematic data were tracked in Qualisys Track Manager (version 2018.1, Qualisys AB, Goteborg, Sweden), and then, both sEMG and kinematic data were imported into Visual3D (version 2020.07.4, C-Motion Inc., Germantown, MD, USA) for further signal processing and data analysis. Kinematic data were interpolated (maximum gap: 10 frames) and low-pass filtered (Butterworth 4th order) with a cut-off frequency of 12 Hz, as determined using residual analysis. For each horse, a rigid-body segment model of the right fore- and hindlimb was created—in accordance with the method described by Hobbs et al. [34]—and applied to all dynamic trials from the corresponding horse. Sagittal plane joint angles were calculated based on the static trial using the cardan sequence x, y, z. Flexion/extension was defined as rotation around the segment coordinate system x-axis, with the flexor side defined as caudal for shoulder, carpal, stifle, MTPJ, and MCPJ joints and as cranial for elbow, hip, and tarsal joints. Joint angular velocity was calculated as the first derivative of joint angular displacement. To calculate pro-retraction angles, fore- and hindlimb segments were defined using markers on the proximal end of the scapular spine and the tuber coxae as the respective proximal ends and the corresponding lateral hoof wall marker (approximately over the centre of rotation of the distal interphalangeal joint) as the distal end. Fore- and hindlimb pro-retraction angles were calculated in relation to a body reference segment, defined using a marker placed between the tubera sacrale and a virtual landmark, projected from the tubera sacrale marker to a point directly above the marker on the scapular spine.

Fore- and hindlimb hoof impact and lift-off events were calculated using the method described by Holt et al. [35] and were applied to kinematic and sEMG signals. Successive right hindlimb impact events were used to segment canter strides, irrespective of whether the hindlimb functioned as LdH or TrH. Discrete spatiotemporal variables were calculated for each canter stride, including fore- and hindlimb stance duration, stride duration, and stride velocity, which was calculated as the first derivative of the tubera sacrale marker coordinates within the laboratory coordinate system (y-axis) averaged over each canter stride. To correct for conformational differences between horses, joint angle data were normalised to the corresponding joint angles from each horse’s static trial and are thus presented as angular changes from the standing position [36,37].

#### 2.4.2. sEMG Data Processing and Analysis

Raw sEMG signals were differentially amplified by a factor of 909, a CMRR of >80 dB and internal Butterworth high-pass (20 ± 5 Hz cut-off, >40 dB/dec) and low-pass filters (450 ± 50 Hz, >80 dB/dec). During post-processing, signals were DC-offset removed, high-pass filtered using a Butterworth 4th order filter with a 40 Hz cut-off frequency [28,38], and full-wave rectified. The ARV was calculated from full-wave-rectified signals from each muscle, using stride duration as the time interval, and normalised relative to the maximum ARV value observed across all strides within each horse and muscle [28]. Prior to normalisation, outlier ARV data were detected and removed using the method described by St. George et al. [31]. Muscle activity onset and offset events were calculated using the double threshold method for equine sEMG signals [31]. The timing threshold was defined as 5% of the average stride duration across all horses, and the amplitude threshold was defined as 5% of the peak amplitude of each individual sEMG signal. Onset and offset events and the resultant activity duration for each muscle were normalised to the percentage of each respective canter stride duration. Timing of peak amplitude for each stride was detected from enveloped (10 Hz) signals and normalized to the percentage of each respective canter stride [31].

For the analysis of continuous sEMG data, full-wave-rectified signals were enveloped using a Butterworth low-pass filter (4th order, 25 Hz cut-off) and normalised to a reference voluntary contraction (RVC). The RVC was defined as the maximum sEMG amplitude value observed across all canter strides within each horse and muscle. Prior to normalisation, peak amplitude values from each canter stride were checked for outliers to ensure that sEMG signals were normalised to a value that accurately reflected the maximum activity observed during canter [31]. The normalisation techniques for ARV and continuous sEMG data permitted examination of the proportional difference between leading and trailing limb muscle function.

### 2.5. Statistical Analysis

Descriptive statistics (mean ± SD) were calculated for discrete kinematic and sEMG variables within each limb. sEMG data from the triceps and splenius were grouped according to the LdF and TrF movement cycles, with the biceps and gluteal grouped according to LdH and TrH movement cycles. Differences in discrete measurements between leading and trailing fore- and hindlimbs (LdH/LdF vs. TrH/TrF) were analysed using within-subjects linear mixed effects models, with limbs modelled as fixed factors and with random intercepts by participants included, whilst adopting the restricted maximum likelihood method. Linear mixed effects models were undertaken using SPSS software (version 28.0.1.1. (15), IBM Corp., Armonk, NY, USA). In addition, one-dimensional Statistical Parametric Mapping (SPM) was used to analyse differences between leading and trailing fore- and hindlimbs using continuous, time-series data from normalised sEMG, joint angle, and joint angular velocity data, which were time normalised to 101 data points per gait cycle. SPM was undertaken within MATLAB 2019b (MATLAB, MathWorks, Natick, USA), using source code available at http://www.spm1d.org/ (accessed on 16 April 2023).

## 3. Results

### 3.1. Kinematic Differences between Leading and Trailing Fore- and Hindlimbs

Kinematic and sEMG data from 67 left-lead and 64 right-lead canter strides were analysed and are presented here. Descriptive statistics for discrete kinematic data are presented as mean ± SD in Table 1. Forelimb stance duration was significantly longer for the TrF compared to the LdF (*p* < 0.05). Stride duration, stride velocity, and hindlimb stance duration did not significantly differ between canter leads and the measured leading and trailing limbs.

**Figure 1:**
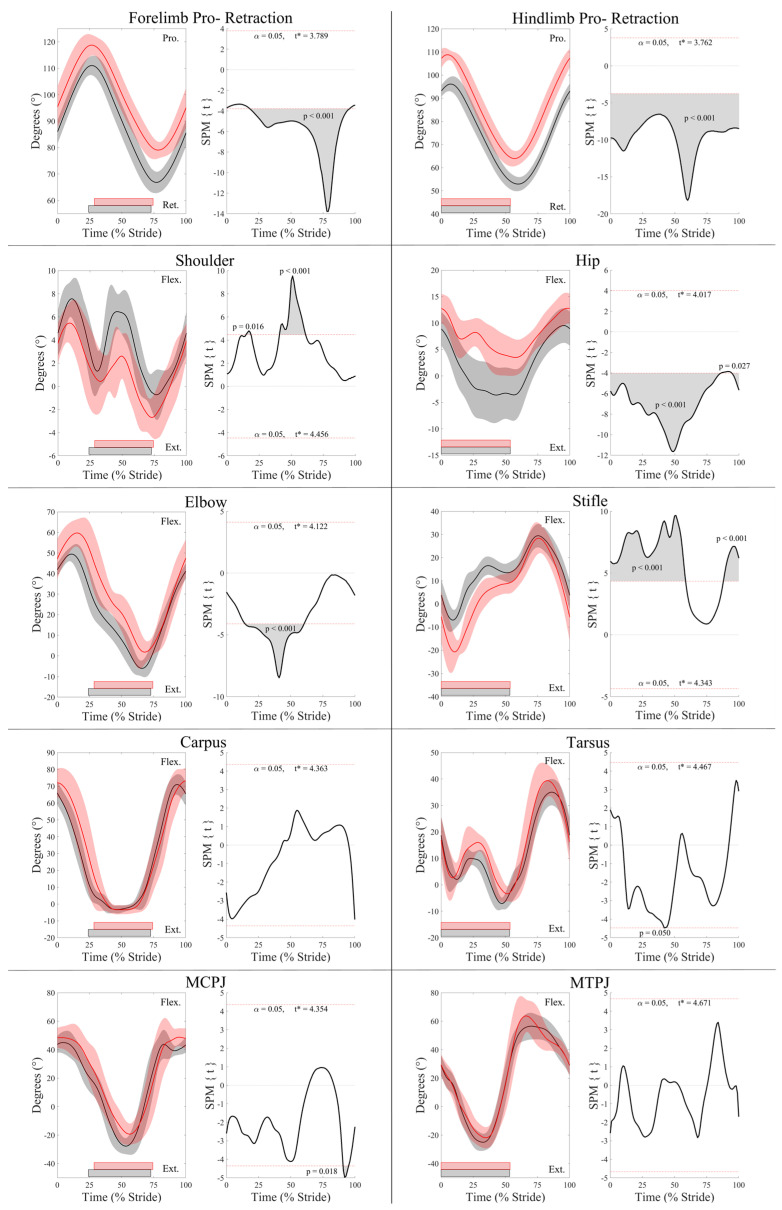
SPM results for normalized sagittal plane joint angles (°) from the leading (red) and trailing (black) fore- and hindlimbs across the group of horses (*n* = 10). For each kinematic variable, left-side graphs illustrate mean (solid line) and standard deviation (shaded area) data, and right-side graphs illustrate the paired samples *t*-test SPM results (black solid line) and the critical thresholds (α, t*) for significance (red dashed line), with grey shaded areas indicating regions/data clusters with statistically significant differences between limbs. *p* values for each data cluster are presented. The joint angle graphs include horizontal bars that represent stance phase duration from their respective leading (red bars) and trailing (black bars) limbs. Data are time-normalized to stride duration, calculated using corresponding impacts of the leading or trailing hind limb.

**Figure 2:**
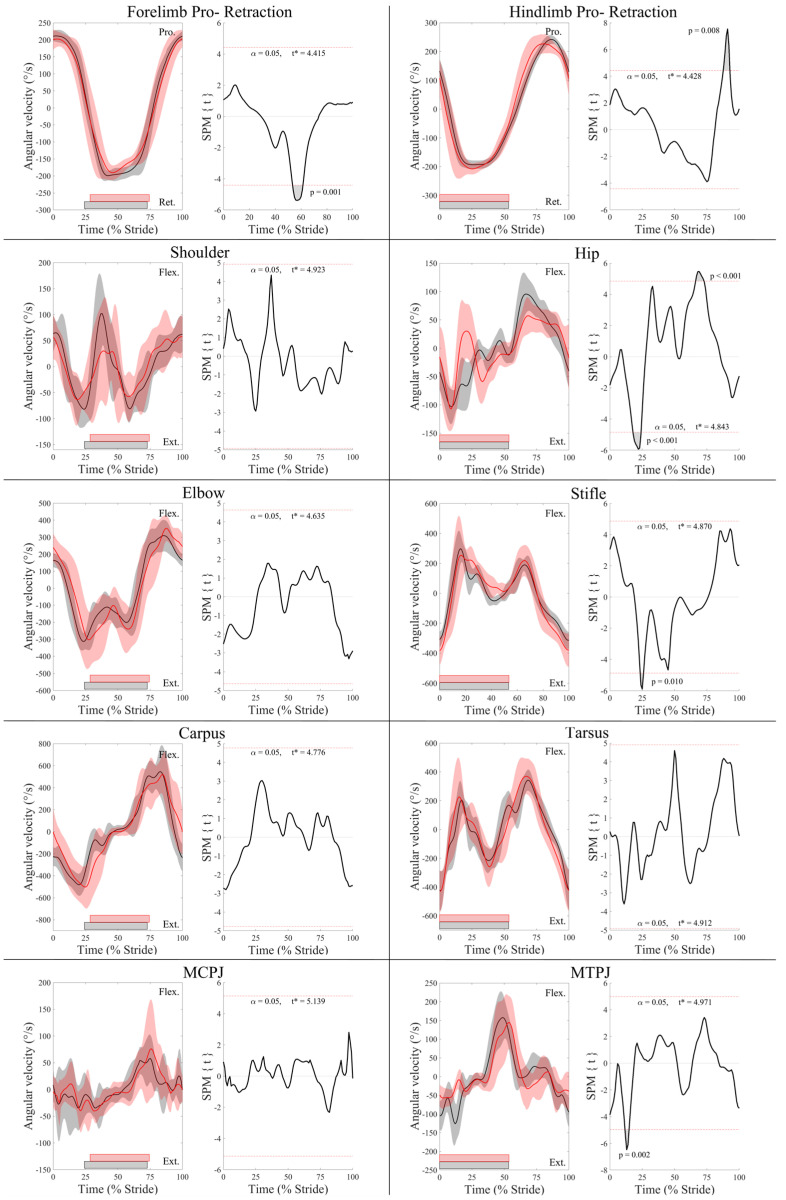
SPM results for normalized sagittal plane joint angular velocity (°/s) from the leading (red) and trailing (black) fore- and hindlimbs across the group of horses (*n* = 10). For each kinematic variable, left-side graphs illustrate mean (solid line) and standard deviation (shaded area) data and right-side graphs illustrate the paired samples *t*-test SPM result (black solid line) and the critical thresholds (α, t*) for significance (red dashed line), with grey-shaded areas indicating regions/data clusters with statistically significant differences between limbs. *p* values for each data cluster are presented. The joint angular velocity graphs include horizontal bars that represent stance phase duration from their respective leading (red bars) and trailing (black bars) limbs. Data are time-normalized to stride duration, calculated using corresponding impacts of the leading or trailing hindlimb.

Group-averaged joint angle–time curves and SPM results for joint angle and joint angular velocity data from the fore- and hindlimbs are presented in Figure 1 and Figure 2, respectively. In the forelimbs, the LdF was more protracted and less retracted than the TrF (*p* < 0.001), and the TrF elbow joint was more extended in late stance (*p* < 0.001). The MCPJ was less flexed during mid-swing (*p* = 0.02) and the elbow was more extended during late swing (*p* < 0.001) in the TrF compared to the LdF. The shoulder angle was more flexed in the TrF than the LdF during the late swing phase (*p* = 0.016) and during the majority of stance (*p* < 0.001). SPM results for forelimb joint angular velocity data (Figure 2) showed no significant differences between the LdF and TrF (*p* > 0.05) except for peak protraction velocity, which was faster in the TrF than the LdF (*p* < 0.001).

As with the forelimbs, in the hind limbs, the LdH was more protracted and less retracted than the TrH (*p* < 0.001) (Figure 1). The TrH hip joint was more extended than the LdH throughout stance (*p* < 0.001) and less flexed throughout swing (*p* < 0.05). In contrast, the stifle joint was more flexed in the TrH than the LdH in late swing (*p* < 0.001) and during stance (*p* < 0.001). In late stance, peak tarsal extension was greater in the TrH than the LdH (*p* = 0.05). Peak angular velocity of hind limb retraction and hip extension were faster for TrH than LdH in late swing (*p* < 0.01) (Figure 2). The TrH exhibited a short period of significantly faster angular velocity of hip, stifle, and MTPJ extension at approximately mid-stance (*p* < 0.05) (Figure 2).

### 3.2. Surface Electromyography Data

**Figure 3:**
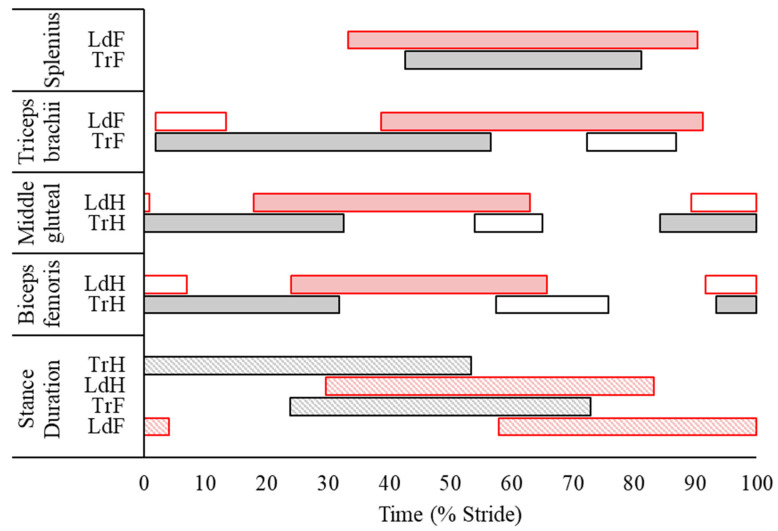
Average phasic muscular activation patterns of the studied muscles across *n* = 10 horses during the canter stride cycle, calculated using average sEMG activity onset and offset events from the leading (LdF) and trailing (TrF) forelimbs, and the leading (LdH) and trailing (TrH) hind limbs. Data are time-normalized to canter stride duration, calculated using impacts of the TrH. To present data from a complete stride cycle, a composite stride was constructed by time shifting the leading (LdF/LdH) limb data using the time ratio between TrH–LdH advanced placement and TrH stance duration (55.5%) presented by Clayton [9] for overground, medium canter. Red and black horizontal bars represent stance phase duration (diagonal stripe fill) and muscle activity duration from the leading (LdF, LdH) and trailing (TrF, TrH) fore- and hindlimbs, respectively. Solid horizontal bars represent the consistent, main burst of muscular activity, whereas unfilled horizontal bars represent intermittent muscular activity across measured strides.

**Figure 4:**
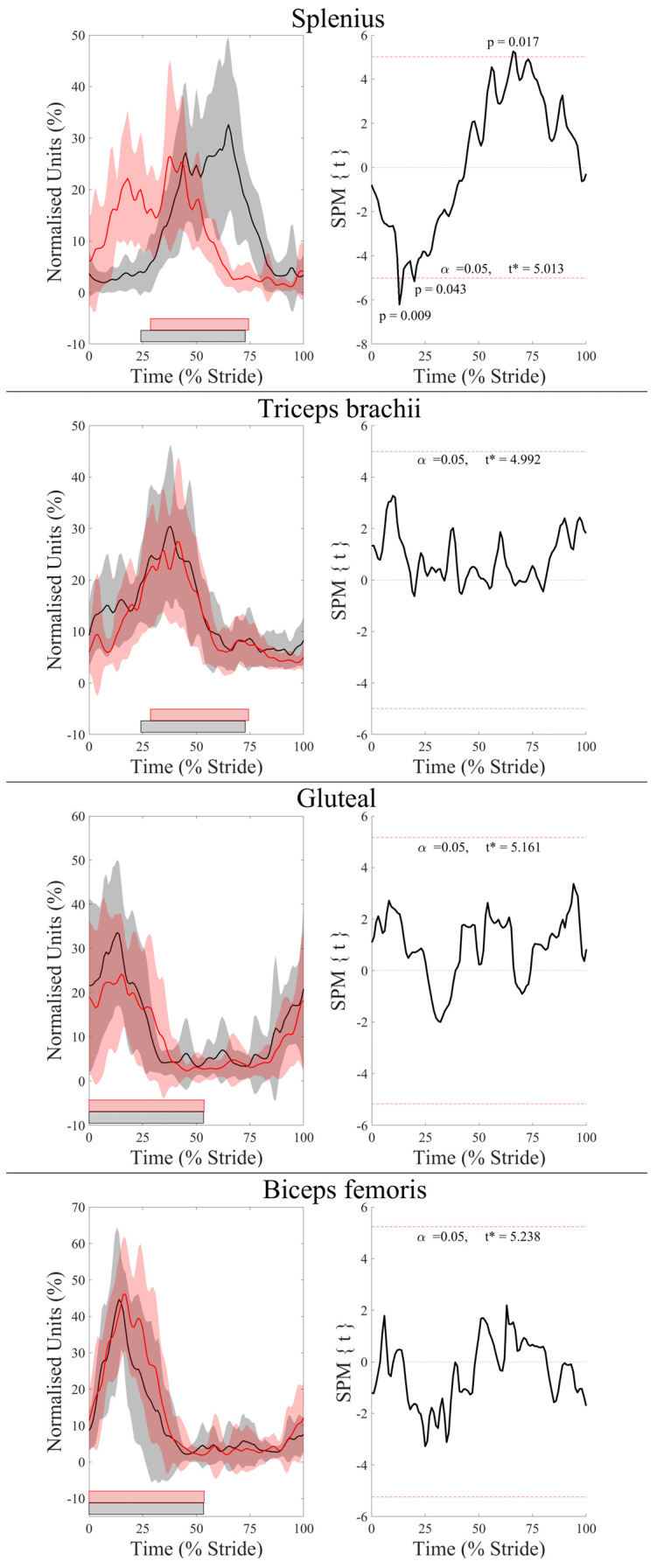
SPM results for time- and amplitude-normalized sEMG data across the group of horses (*n* = 10) for studied superficial muscles of the leading (red) and trailing (black) fore- and hindlimbs across the group of horses (*n* = 10). Left-side graphs illustrate mean (solid line) and standard deviation (shaded area) sEMG data, and right-side graphs illustrate the paired samples *t*-test SPM result (black solid line) and the critical thresholds (α, t*) for significance (red dashed line), with grey shaded areas indicating regions/data clusters with statistically significant differences between conditions. *p* values for each data cluster are presented. Horizontal bars represent stance phase duration from the respective leading (red bars) and trailing (black bars) limbs. Data are time-normalized to stride duration, calculated using corresponding impacts of the leading or trailing hindlimb.

Descriptive statistics for discrete sEMG data are presented as mean ± SD in Table 2. In addition, the average phasic activation patterns of each muscle—derived from sEMG activity onset and offset events—together with the stance phases of the leading and trailing fore- and hindlimbs are illustrated in Figure 3. In the forelimbs, discrete (Table 2, Figure 3) and continuous sEMG data (Figure 4) from triceps did not significantly differ between the LdF and TrF (*p* > 0.05). There was a significant phasic shift in the splenius activation pattern, in which LdF activity onset, offset, and peak amplitude occurred earlier in the stride cycle than TrF (*p* < 0.05), with non-significant differences in ARV and activity duration observed between limbs (*p* > 0.05) (Table 2, Figure 3). This phasic shift in splenius activation was observed in continuous sEMG waveforms, where SPM results (Figure 4) showed significant differences in splenius activity during late-swing and late-stance phase of the forelimbs (*p* < 0.05). In the hindlimbs, ARV and activity duration of the TrH gluteal were greater (*p* < 0.05) than the LdH (Table 2, Figure 3). Activation offset of the LdH biceps occurred later in the stride cycle than the TrH (*p* < 0.05), but activity duration was not significantly affected by this (*p* > 0.05) (Table 2, Figure 3). SPM results for hindlimb muscles showed no significant differences between the LdH and TrH (*p* > 0.05) (Figure 4).

## 4. Discussion

In this study, we combined sEMG and motion capture to conduct the first comparative study of muscle activation and movement within the leading and trailing fore- and hindlimbs during ridden, overground canter. In the forelimbs, the TrF showed greater shoulder flexion and greater elbow extension in midstance, but the triceps muscle, which acts across both joints, showed a non-significant trend for greater ARV and activity duration within this limb. Thus, our hypothesis that the TrF would exhibit greater joint flexion/extension during stance phase and greater muscular activity across the stride cycle than the LdF can be partially accepted. We also investigated splenius activation in the context of LdF and TrF movement cycles and found a significant phasic shift for earlier activation within the LdF stride cycle, with non-significant differences in ARV and activity duration observed between limbs. In the hindlimbs, we observed significantly greater LdH protraction and hip flexion during swing and significantly greater stifle extension during stance but with significantly less gluteal activity across the stride cycle compared to the TrH. Thus, our hypothesis that the LdH would exhibit greater joint flexion/extension and muscular activity across the stride than TrH was also partially accepted.

### 4.1. Electromyographic and Kinematic Differences between Leading and Trailing Forelimbs

In comparison to the LdF, the more vertically loaded TrF [5,17] exhibited a significantly greater retraction angle, with greater shoulder flexion and elbow extension during stance. In contrast, the LdF had significantly greater protraction driven by significantly greater elbow flexion during swing. These kinematic findings agree with the between-limb differences reported in previous studies of horses cantering overground [16] and on a treadmill [4] at faster speeds than were studied here. Although we observed significantly greater MCPJ flexion in the LdF during swing, which agrees with these studies [4,16], they also reported significantly greater maximal MCPJ extension in the TrF during stance, which was observed in our data but did not reach statistical significance. Since TrF is the limb with the highest peak vertical force at canter [5,15,17], the significantly longer TrF stance duration that was observed here but not in a previous study [4] may be a strategy to provide the necessary impulse over a longer time with a lower peak load [17,39]. This may suggest that, at the speed studied here, differences in limb loading were modulated by significant alterations in stance duration and non-significant alterations in peak MCPJ extension, which exhibits a linear relationship with limb force [18]. However, further research using ground reaction force (GRF) data is required to confirm this. As such, methodological differences—in particular, differences in canter speed and the study of overground vs. treadmill locomotion—may explain discrepancies in temporal findings and MCPJ kinematics between studies.

Crevier-Denoix et al. [16] is the only known study to measure and compare joint angular velocity of the forelimbs during canter measured on turf and synthetic surfaces, but only reported data from the MCPJ and carpal joints. In accordance with this study, they found no significant differences in carpal joint angular velocity or peak MCPJ extension velocity during stance on either surface but reported significantly greater peak MCPJ flexion velocity of the LdF than the TrF during late stance, on the turf surface only [16]. In agreement with Crevier-Denoix et al. [16], we did not observe significant differences in MCPJ flexion velocity during canter on a synthetic surface. Thus, despite some discrepancies in temporal findings and MCPJ kinematics between studies, our overall findings agree with previous research comparing kinematics of the LdF and TrF during canter [4,16] and corroborate their functional roles as the “swinging” and “supporting” limbs, respectively [4].

Harrison et al. [19] noted that, during walk and trot, most FL muscles display peak EMG activity at the hoof impact event but noted two different peaks at mid-stance and in early swing during canter. This agrees with phasic activity patterns observed in this study for the triceps, in which a main burst of activity was consistently observed from late-swing to mid-late stance, with a second shorter and less frequently observed burst following FL lift-off (Figure 3). Thus, our findings support the suggestion by Harrison et al. [19] that this additional burst at the beginning of swing may reflect the need for active muscle contraction to aid passive forces, produced by the tendons and ligaments, in the generation of greater joint torques that are required to sustain a steady canter gait. Triceps activity did not differ significantly between LdF and TrF, but subtle differences in sEMG amplitude and phasic activation patterns appear to reflect the facilitation of significantly different movement cycles between forelimbs. Earlier, albeit non-significant, triceps activation within the TrF corresponded with significantly earlier and decreased peak limb protraction and elbow flexion and earlier hoof impact, and vice versa in the LdF. Non-significant differences in ARV may also reflect the differing functions of each limb during stance phase, where triceps activation is possibly more related to stabilization of the shoulder and elbow joints against the greater vertical forces experienced by the TrF and against the high braking forces experienced by the LdF [5,15]. We suggest that future studies examine co-contraction of the biceps brachii and triceps brachii, which has previously been described as important for joint stability, positional control of the limb, and mitigation of shear loading during stance phase at canter [19]. Therefore, studies examining the synergistic activity of biceps brachii and triceps brachii may provide further insight into the neuromuscular strategies of the LdF and TrF during canter.

We observed significantly different phasic activity patterns of the splenius between the LdF and TrF movement cycles, offering some insight into splenius activity in relation to ipsilateral forelimb movement during canter. Splenius activity onset occurred from mid-swing to mid-stance phase during the LdF movement cycle and from early-stance to early-swing phase during the TrF movement cycle (Figure 3). Tokuriki and Aoki [40] noted the same asymmetrical phasic activation pattern for splenius relative to the ipsilateral forelimbs during canter, measured using intramuscular EMG. They summarised that the splenius is bilaterally activated during the late-swing phase of the TrF, just prior to impact [40]. This agrees with the activity onset detected here, in which the muscle remained active during the diagonal support phase of TrF and LdH. At this point in the stride cycle, sagittal pitching of the trunk and neck segments are largely “in phase”, resulting in a “nose down”—or clockwise—pitching angle [7,29,30], which occurs during 50% of the stride cycle and corresponds to the activity duration of the splenius observed here, supporting previous suggestions that the splenius is bilaterally activated to counteract the downward movement of the head and neck [7]. In the remaining 50% of the canter stride, which includes the suspension phase and the single limb support phases of the LdF and TrH, sagittal pitching of the trunk and neck segments are “out of phase” and the neck segment is elevated in a “nose up” or counterclockwise position [7,29,30]. During this time, Gellman et al. [29] reported that active muscular work is required to reverse downward angular rotation and raise the head and neck, after which the nuchal ligament passively provides most of the mechanical work required to elevate the head and neck through the release of elastic strain energy. Our findings support this claim, as splenius activation was not observed during the single support and suspension phases. However, splenius activity observed during LdF stance may represent the active muscular contribution for reversing the neck segment rotation [29], particularly during the high decelerating forces experienced by this limb [5,15], which together may contribute to vertical lifting of the COM for the upcoming suspension phase [30]. Further studies are required to investigate the relationship between splenius activity and head and neck kinematics at canter, but our findings provide objective support for the splenius making an active contribution to the characteristic body pitching mechanisms that occur during this gait [7,29,30].

### 4.2. Electromyographic and Kinematic Differences between Leading and Trailing Hindlimbs

Kinematic findings from the hindlimbs corroborate a previous report of greater LdH protraction and greater TrH retraction during canter on a treadmill [4]. These were associated with significantly greater LdH hip joint flexion during swing and significantly greater TrH hip joint extension during stance. Additionally, Back et al. [4] reported significantly greater peak MTPJ extension in TrH and peak tarsal flexion in LdH during stance, which was also observed here but did not reach statistical significance when analysed using SPM. In contrast to Back et al. [4], who observed significantly greater TrH stifle flexion only during early stance, our evaluation of continuous joint angle data revealed significantly more flexion of the TrH stifle throughout stance and the majority of swing. Again, discrepancies for variables reaching significance may be due to methodological differences between studies, as described above. In particular, the use of SPM in this study allows comparison between continuous angle–time waveforms to statistically compare, for the first time, the limb movements across the entire canter stride cycle, whereas previous kinematic investigations of canter compared discrete kinematic variables between limbs [4,16]. Furthermore, to reduce inter- and intra-subject variability, we normalised joint angle–time data to the standing position, which was not performed in the other comparative studies [4,16] and may account for some discrepancies.

The significantly greater and delayed peak protraction velocity of TrH during swing is probably related to the peak retraction angle occurring significantly later, necessitating faster protraction during late swing to enable correct limb positioning for impact. During early stance, the MTPJ extended more rapidly in the TrH than the LdH, suggesting that the TrH is more rapidly loaded [18] and/or generates propulsive forces more rapidly than the LdH [5]. This significant peak in TrH MTPJ extension velocity was followed by significant peaks in extension velocity of the hip and stifle joints that were not present in the LdH and occurred at approximately mid-stance, which coincided with significantly greater TrH hip joint extension and stifle joint flexion. Taken together, these findings suggest that the proximal joints of the TrH actively damp vertical loading, as has been observed as a compensatory strategy in lame horses [41]. Integration of GRF and kinematic data is required to confirm this, but our kinematic results from the hindlimbs corroborate the propulsive and supporting functions of the TrH and LdH, respectively [4,5,15].

A main burst of activation from late swing to approximately mid-stance was observed for the middle gluteal and biceps femoris muscles during canter, which agrees with previous equine EMG studies [23,42,43,44]. Muscle activity during the first half of stance is related to limb loading and the generation of positive work, primarily by the hip joint [45], which is stabilized by contraction of the middle gluteal and hamstring muscles [46]. Quiescent activity during late stance phase and the majority of swing phase, appears to reflect a passive contribution of the gluteal and biceps to hip flexion and limb protraction, supporting the highly economical locomotor strategy of horses, which has been described for faster gaits, such as canter [47]. We observed significantly greater ARV and significantly longer activity duration for the TrH gluteal compared to LdH. This finding may be related to the generation of greater muscular force for stabilizing the more extended TrH hip joint during stance [46] and to produce the large propulsive forces required to generate a strong push-off for the rapid reversal of COM movement during stance [5]. Like forelimb movement and triceps activity, earlier—albeit non-significant—onset of gluteus activity in the TrH coincided with the significantly earlier initiation of hip extension and greater limb retraction velocity in late swing. This suggests an active muscular contribution to earlier reversal of this limb’s direction of movement.

In contrast to the gluteal, significant between-limb differences were not observed for biceps activity, except for a significantly delayed activation offset in the LdH. St. George et al. [28] reported significantly greater ARV for the LdH biceps when appropriate sEMG signal processing and analysis procedures were employed. The same recommended procedures were employed here, but our findings did not reach statistical significance for ARV. Mean difference values between limbs were similar between studies at approximately 20% (17.5% vs. 21.5% reported by [28]), but we observed greater SD values, which may account for non-significant findings for ARV in this different group of horses. Still, the differing roles of the LdH and TrH are shown by the significantly greater TrH gluteal activity that stabilises the more extended hip joint and generates the highest propulsive forces across all limbs at canter [5,15]. In the LdH, greater—albeit non-significant—biceps activity may reflect its role for stabilising the hip, stifle, and hock joints against the greater decelerative and vertical forces experienced by this limb [5].

### 4.3. Practical Applications for Equestrian Training

The canter is an essential gait for equestrian competition and training, particularly for dressage and jumping disciplines, which aim to develop an equine athlete that exhibits straightness, balance, and symmetry of gait [4,10,48,49]. However, it is widely accepted within the equestrian and scientific community that horses display a motor asymmetry that can manifest as a preference to use one canter lead over the other [48,49]. As such, attempts to correct the “sidedness” or motor asymmetry of a horse is central to most equestrian training programmes [48,49,50]. In the scientific literature, there is contention surrounding whether motor asymmetry is inherent, the result of cerebral lateralisation, and/or linked to external factors, such as asymmetrical training or rider position/laterality [49,51]. Here, we have provided objective evidence for the differing functional demands of the studied muscles when they act within the leading and trailing limbs during canter. A deficiency in muscular strength on one side of the horse’s body may result in one canter lead being more physically demanding and could thus offer another factor contributing to a horse’s preference to use a specific canter lead. As such, our findings provide objective evidence for training the horse equally on both canter leads to develop symmetry in muscular strength, enhance athletic performance, and mitigate the risk of overuse injuries. In addition, an understanding of the differing neuromuscular demands between leading and trailing limbs during canter may allow trainers to better identify muscular imbalances and to implement exercises to improve muscular symmetry during canter. These exercises may include, but are not limited to, lateral movements [49,51] and/or working the horse at various speeds and inclines, each of which have been shown to elicit modifications in muscular activation or workload [26,42,43,52,53].

### 4.4. Study Limitations

Kinematic and sEMG data were collected unilaterally from the right side of the horses, so movement and muscle activity of the leading and trailing limbs could not be compared within the same stride. Furthermore, the measurement of one hind limb meant that impact of either the LdH or TrH was used for stride splitting depending on the canter lead, which may confound comparisons of temporal stride characteristics between leading and trailing limbs. This method did, however, allow direct comparison of movement and muscle activity when the same limb functioned as the leading or trailing limb as in previous studies of asymmetrical gaits [4,16,26]. In addition, we studied a relatively equal distribution of 67 left- and 64 right-lead canter strides, which permitted direct comparisons between limbs. When researchers acquire bilateral kinematic and sEMG data during canter, they should consider the potential for sEMG asymmetry due to electrode placement and underlying tissue differences between left- and right-side muscles [54], which our study design mitigated.

Canter speed, which was not standardised, is known to affect muscle activity and kinematics [55]. However, speed did not differ between left- and right-lead canters, and standard deviation values for this variable were relatively low compared to other studies of ridden, overground canter [6,9,16]. Horses were ridden by their normal rider, so the rider’s influence was not standardised. Interestingly, Tokuriki and Aoki [40,44,56] reported that the rider did not influence the phasic activation patterns of intramuscular EMG signals from biceps femoris, triceps brachii, and splenius during canter when compared to unridden conditions. In addition, the joint angle–time diagrams and kinematic findings reported here were similar to a study of unridden horses that standardised canter speed using a treadmill [4]. As such, we suggest that our findings are externally valid within the context of ridden horse training and performance. Finally, future work with larger numbers of horses is required to study differences in leading and trailing limbs within the wider population.

## 5. Conclusions

This study combined sEMG and motion capture to conduct the first comparative study of muscle activation and movement within the leading and trailing fore- and hindlimbs during ridden, overground canter. We observed significantly greater protraction in the LdF and LdH through respectively greater elbow and hip flexion during swing phase when compared to the trailing limbs, but these differences were not associated with significant differences in triceps activity between LdF and TrF. In the trailing limbs, we observed significantly greater retraction than the leading limbs during stance, driven by significantly greater TrF elbow and TrH hip joint extension. In the hindlimbs, this difference was associated with significantly greater gluteal ARV and activity duration in the TrH, which reflects the requirement for greater muscular force to stabilize the more extended TrH hip joint during stance and to produce large propulsive forces. We also observed a significant phasic shift for earlier splenius activation within the LdF stride cycle, which reflected bilateral activation to counteract the downward movement of the head and neck during TrF and LdH diagonal support. Our findings provide novel insight into the underlying alterations in muscle activation that facilitate the differing biomechanical functions of the leading and trailing, fore- and hind limbs, as well as the splenius’ active contribution to the characteristic body pitching mechanisms in cantering horses. These findings have real world applications for equestrians, as they provide objective justification for exercising the horse equally on both canter leads to promote balanced muscular development and to mitigate the risk of overuse injury.

## Figures and Tables

**Table 1:** Mean ± standard deviation for stride velocity (m/s) and measured temporal kinematic variables. Data are grouped according to left and right canter lead, where the measured limbs function as TrF/TrH and LdF/LdH, respectively. Differences between canter leads and the associated leading and trailing limbs are presented for each variable as *p* values.

Variable	Canter Lead/Limb		*p* Value
	Left Lead TrH or TrF	Right Lead LdH or LdF	
Stride velocity (m/s)	4.41 ± 0.42	4.42 ± 0.40	0.729
Stride duration (s)	0.59 ± 0.02	0.59 ± 0.02	0.641
Forelimb stance duration (s)	0.29 ± 0.02	0.27 ± 0.02	0.018 *
Hindlimb stance duration (s)	0.32 ± 0.03	0.32 ± 0.04	0.786

* Between-limb differences are significant at the 0.05 level. Abbreviations: leading forelimb (LdF), leading hindlimb (LdH), trailing forelimb (TrF), trailing hindlimb (TrH).

**Table 2:** Mean ± standard deviation for discrete sEMG variables from each superficial muscle of interest. Data are grouped according to leading (LdF/LdH) and trailing (TrF/TrH) fore- and hindlimbs. Differences between limbs are presented for each variable as *p* values.

Muscle	Variable	Limb		*p* Value
		TrH	LdH	
Biceps femoris	ARV (%)	62.7 ± 20.3	80.2 ± 14.9	0.179
	Activity duration (% stride)	46.9 ± 13.7	47.5 ± 14.9	0.822
	Peak amplitude (% stride)	16.5 ± 5.9	16.7 ± 7.2	0.541
	Activity offset (% stride)	31.9 ± 7.8	36.2 ± 6.5	0.018 *
	Activity onset (% stride)	93.5 ± 6.5	94.3 ± 5.7	0.287
Middle gluteal	ARV (%)	77.6 ± 14.1	66.3 ± 19.4	0.046 *
	Activity duration (% stride)	54.8 ± 13.8	48.6 ± 14.5	0.024 *
	Peak amplitude (% stride)	13.6 ± 5.2	16.7 ± 8.2	0.163
	Activity offset (% stride)	32.6 ± 7.5	33.5 ± 7.5	0.340
	Activity onset (% stride)	84.2 ± 7.3	88.2 ± 9.6	0.069
		**TrF**	**LdF**	
Triceps brachii	ARV (%)	72.5 ± 18.2	68.5 ± 19.7	0.179
	Activity duration (% stride)	66.4 ± 14.4	56.5 ± 18.0	0.234
	Peak amplitude (% stride)	40.4 ± 13.9	35.9 ± 13.7	0.531
	Activity onset (% stride)	101.8 ± 9.4 ^§^	109.1 ± 9.7 ^§^	0.061
	Activity offset (% stride)	56.6 ± 6.5	56.6 ± 9.9	0.368
Splenius	ARV (%)	75.8 ± 17.0	64.8 ± 17.7	0.322
	Activity duration (% stride)	51.1 ± 10.3	57.2 ± 12.5	0.192
	Peak amplitude (% stride)	59.0 ± 11.4	32.3 ± 15.2	0.000 *
	Activity onset (% stride)	42.7 ± 25.9	103.7 ± 14.0 ^§^	0.002 *
	Activity offset (% stride)	81.2 ± 7.0	60.7 ± 10.0	0.029 *

* Between-limb differences are significant at the 0.05 level. Abbreviations: average rectified value (ARV), leading forelimb (LdF), leading hindlimb (LdH), trailing forelimb (TrF), trailing hindlimb (TrH). Note that only activity onset and offset events from the main activation burst (as presented in Figure 3) of each muscle are presented here. ^§^ Values > 100% reflect activity onsets that spanned the end to the beginning of the stride cycle and so activity onsets < 20% for these muscles were normalized to permit direct comparisons between horses, strides, and limbs.

## Data Availability

The data presented in this study are openly available in UCLanData at https://doi.org/10.17030/uclan.data.00000371 (accessed on 9 May 2023), reference number 371.
